# NPM-ALK: A Driver of Lymphoma Pathogenesis and a Therapeutic Target

**DOI:** 10.3390/cancers13010144

**Published:** 2021-01-05

**Authors:** Elissa Andraos, Joséphine Dignac, Fabienne Meggetto

**Affiliations:** 1 Inserm, UMR1037 CRCT, F-31000 Toulouse, France; elissa.andraos@inserm.fr (E.A.); josephine.dignac@univ-tlse3.fr (J.D.); 2 Université Toulouse III-Paul Sabatier, UMR1037 CRCT, F-31000 Toulouse, France; 3 CNRS, ERL5294 CRCT, F-31000 Toulouse, France; 4 Institut Carnot Lymphome-CALYM, F-31024 Toulouse, France; 5 Laboratoire d’Excellence Toulouse Cancer-TOUCAN, F-31024 Toulouse, France

**Keywords:** lymphoma, anaplastic large cell lymphoma, tyrosine kinase, anaplastic lymphoma kinase, chemotherapy, target therapy, drug resistance

## Abstract

**Simple Summary:**

Anaplastic lymphoma kinase (ALK) is a tyrosine kinase associated with Anaplastic Large Cell lymphoma (ALCL) through oncogenic translocations mainly NPM-ALK. Chemotherapy is effective in ALK(+) ALCL patients and induces remission rates of approximately 80%. The remaining patients do not respond to chemotherapy and some patients have drug-resistant relapses. Different classes of ALK tyrosine kinase inhibitors (TKI) are available but used exclusively for EML4-ALK (+) lung cancers. The significant toxicities of most ALK inhibitors explain the delay in their use in pediatric ALCL patients. Some ALCL patients do not respond to the first generation TKI or develop an acquired resistance. Combination therapy with ALK inhibitors in ALCL is the current challenge.

**Abstract:**

Initially discovered in anaplastic large cell lymphoma (ALCL), the ALK anaplastic lymphoma kinase is a tyrosine kinase which is affected in lymphomas by oncogenic translocations, mainly NPM-ALK. To date, chemotherapy remains a viable option in ALCL patients with ALK translocations as it leads to remission rates of approximately 80%. However, the remaining patients do not respond to chemotherapy and some patients have drug-resistant relapses. It is therefore crucial to identify new and better treatment options. Nowadays, different classes of ALK tyrosine kinase inhibitors (TKI) are available and used exclusively for EML4-ALK (+) lung cancers. In fact, the significant toxicities of most ALK inhibitors explain the delay in their use in ALCL patients, who are predominantly children. Moreover, some ALCL patients do not respond to Crizotinib, the first generation TKI, or develop an acquired resistance months following an initial response. Combination therapy with ALK inhibitors in ALCL is the current challenge.

## 1. Introduction

In 1994, the anaplastic lymphoma kinase (ALK), a receptor tyrosine kinase, was initially identified by Steve Morris through its involvement in the t(2;5)(p23;q35) chromosomal translocation associated with anaplastic large cell lymphoma (ALCL), a subset of T-cell lymphomas [1]. This fusion combines the cytoplasmic domain of ALK to the N-terminus of nucleolar phosphoprotein 1 (NPM1). Three years later, in 1997, the full-length human anaplastic lymphoma kinase (ALK) receptor or CD246 was entirely sequenced [2]. Although its normal physiological role is not entirely clear, ALK seems to play a role in the development of the nervous system based on its high level of expression in embryonic neural tissue.

The full-length ALK cDNA was originally cloned from the Rh30 rhabdomyosarcoma cell line and its expression has been confirmed in the alveolar subtype of primary rhabdomyosarcoma [3].

The human *ALK* gene is located at the chromosome region 2p23.2–p23.1, contains 26 exons and encodes the full-length ALK protein which includes 1620 amino acids and weighs 180 kDa. N-linked glycosylation is a highly regulated post-translational modification, which is involved in several biological processes such as protein folding and conformation, oligomerization, sorting, cell-cell interactions, and targeting of proteins to sub- or extra-cellular locations. As a result of the 16 N-glycosylation sites in ALK, the molecular weight of the full-length ALK is of approximately 220 kDa [4].

**Figure 1:**
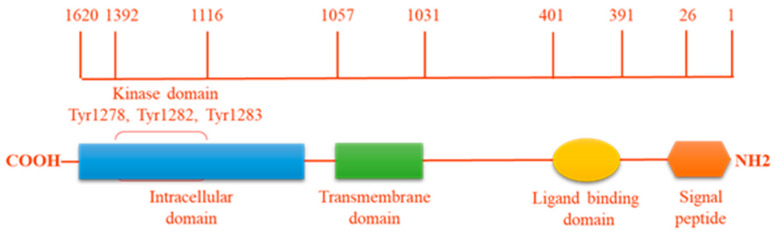
ALK protein structure. The intracellular tyrosine kinase domain harbors the 3 tyrosine motif Tyr1278, Tyr1282, Tyr1283, which represents the major auto-phosphorylation site regulating kinase activity.

ALK is an enzyme with tyrosine kinase activity (Figure 1), which catalyses the transference of a gamma-phosphate group from adenosine triphosphate (ATP) to a tyrosine residue on a substrate protein. One member of the receptor tyrosine kinase (RTK) ALK family contains three parts: an extra-cellular ligand-binding domain (ECD), a single membrane-spanning domain and an intracellular cytoplasmic catalytic domain (ICD). Whilst the tyrosine kinase domain of human ALK shares a high degree of similarity with that of the insulin receptor (IR), its extracellular domain is unique among the RTK family in containing two MAM domains (meprin, A5 protein, and receptor protein tyrosine phosphatase mu), an LDLa domain (low-density lipoprotein receptor class A) and a glycine-rich region [1,5]. Based on the overall homology of the ECD, ALK is closely related to the leukocyte receptor tyrosine kinase (LTK) which also has a glycine-rich region in its ECD. Thus, together with the insulin receptor, ALK forms a unique subgroup in the IR RTK superfamily [4].

During the embryonic development of chickens, mice, rats, and humans, the ALK expression profile is restricted to discrete areas of the developing nervous system and the ALK expression level decreases after birth. ALK has also been identified in non-vertebrate model organisms *Drosophila melanogaster* and *Caenorhabditis elegans* where it is expressed at low levels in the nervous system and is proposed to inhibit or destabilize synapse differentiation. While the function(s) of ALK is not specifically addressed, its conserved expression within the CNS suggests an important role(s) in neural development [6].

Several putative human ALK ligands have been discovered, including pleiotrophin (PTN) and Midkine (MK), two growth factors involved in brain development during embryonic life. However, other studies were unable to prove ALK activation by PTN and MK. Recently, human secreted small proteins ALKAL1 and 2 (for “ALK and LTK ligand”), which were previously reported as family-with-sequence-similarity-150 (FAM150) or Augmentor (AUG), have been shown to bind to both ALK and LTK ECD to activate human ALK and LTK kinase activity in cell culture and when coexpressed in *Drosophila* eyes. FAM150B (or Augmentor alpha or ALKAL2) is a high affinity ALK ligand, compared to Augmentor beta (FAM150A or ALKAL1), from the same family, which binds ALK with poor affinity [7]. In vivo studies using a zebrafish model also revealed ALKALs as ligands of the ALK/LTK receptor family. During fruit fly embryogenesis in *Drosophila*, jelly belly (Jeb has already been discovered as a biological ligand of ALK, activating it to promote gut development by triggering its downstream signaling, such as the ERK signaling pathway. In *Drosophila*, ALK and its ligand Jeb also play a critical role in the development of the visual system. However, the bona fide putative ligand of mammalian ALK would be heparin as a putative heparin-binding motif was found in the N-terminal region of the ALK ECD. Whereas heparins with short chain lengths bound to ALK in a monovalent manner did not activate the receptor, longer heparin chains induced ALK dimerization and activation in cultured neuroblastoma cells. Moreover, antibodies that bound to the extracellular domain of ALK interfered with heparin binding and prevented heparin-mediated activation of ALK [8].

The ALK receptor tyrosine kinase also belongs to the functional family of so-called ‘dependence receptors’. Such receptors function with a dual signaling: when the ligand is bound, the receptor exerts a pro-survival/anti-apoptotic effect on the cell; by contrast, without the ligand ALK becomes proapoptotic [9,10]. Without ligand binding to activate its kinase activity, ALK can be cleaved by caspase-3 during apoptosis (ALK cleavage site (amino acids 1160-1163: DELD), which releases an intracellular ALK fragment (about 60 kDa) into the cytoplasm and exposes a pro-apoptotic segment (addiction/dependence domain, ADD) within the ALK juxtamembrane region, thus amplifying the apoptotic process [11].

In parallel, an alternative transcription initiation (ATI) site in intron 19 of the ALK gene was identified, as well as the existence of three predicted in-frame translation start codons (ATGs) in the ALK ATI transcript, inducing three ALK isoforms (ALK^ATI^) with molecular weights of 61.1, 60.8, and 58.7 kDa. These three ALK isoforms are expressed in approximately 2% to 3% of melanomas and sporadically in several other human cancers, such as lung adenocarcinoma and kidney renal clear cell carcinoma. Moreover, the molecular weights of these ALK isoforms are a little higher than that of the ALK fragment that is released into the cytoplasm after caspase-3 cleavage and ALK ATI isoforms exist in the cytoplasm and the nucleus. The existence of ALK isoforms indicates that ALK may not only function as a membrane binding RTK but also as a cytoplasmic kinase when these active isoforms of ALK are expressed. Indeed, the ALK^ATI^ isoforms, which are kinase active, contain in vitro and in vivo oncogenic capacities and the ALK inhibitor crizotinib inhibited both ALK^ATI^ kinase activity and ALK^ATI^ tumorigenesis ability [12].

In various cancers, other aberrant ALK forms and aberrant ALK expression are generally caused by at least one of four alterations in the *ALK* gene mechanisms: mutations, gain-of-function mutations, amplification, or translocations. The full length ALK receptor is overexpressed by mutations or copy gains in multiple tumor types such as rare cases of B-cell lymphoma [13] and paediatric neuroblastoma [14], glioblastoma, rhabdomyosarcoma, thyroid, and breast cancers [15]. The mechanism of ALK amplification in neuroblastoma is attributable to gene amplification or ALK mutations [16]. *ALK* gene amplification results in increased protein expression and constitutive catalytic activation [17]. Enhancement of the constitutive activation of the tyrosine kinase is also observed when the *ALK* gene is translocated with different partners in solid cancers such as inflammatory myofibroblastic tumors (TPM3-ALK, TPM4-ALK, CLTL-ALK, ATIC-ALK, and CARS-ALK), non-small cell lung cancer (NSCLC: EML4-ALK, TFG-ALK, KIF5B-ALK, KCL1-ALK, and PTPN3-ALK) [18], histiocytic tumors, Spitz tumor, carcinomas (renal: TPM3-ALK, EML4-ALK, and VLC-ALK; colorectal: EML4-ALK and C2orf44-ALK; breast: EML4-ALK) [19], ovarian cancer (FN1-ALK) and in hematopoietic cancers such as diffuse large B-cell Lymphoma (DLBCL: NPM-ALK, CLTC1-ALK, SQSTM1-ALK, and SEC31A-ALK) [20] and Anaplastic Large-Cell Lymphoma (ALCL) (Table 1) [21].

Although as of 2020, over 92 fusion partners have been discovered in ALK(+) NSCLC, in solid cancers, the most common ALK fusion partner is echinoderm microtubule-associated protein-like 4 (EML4), which was found in 6.7% of cases. 46% of ALCL also bear a signature translocation. Indeed, the majority (80%) of ALCL patients bear a t(2;5) translocation which leads to the ectopic expression of a chimeric ALK tyrosine kinase, NPM-ALK also known as p80, since the chimeric protein weighs 80 kDa [15]

Pathologists now have a vast experience in routine immunohistochemical testing (IHC) of the overexpression of the chimeric ALK protein. IHC can also provide information as to the potential ALK fusion partner. Thus, the NPM-ALK chimeric protein is typically detected in the nucleus, nucleolus, and cytoplasm, whereas the majority of other ALK partners (TPM3, TPM4, TFG, ATIC, CTLC) are expressed in the cytoplasm whereas a localization in the periphery of the nucleus is noted in the RANBP2-ALK fusion protein, resulting from the association of the fusion protein with wild-type RANBP2 (a nuclear pore protein), and the moesin (MSN)–ALK fusion protein owing to the association of wild-type MSN and other membrane proteins [22,23,24,25]. Reverse transcriptase-PCR (RT-PCR) best defines the ALK status and detection using RT-PCR of early Minimal Residual Disease (MRD) positivity at diagnosis correlates with a very high relapse risk and lower survival in children with ALK(+) ALCL [26,27,28,29].

## 2. NPM-ALK-Positive Anaplastic Large-Cell Lymphoma

In 2016, the World Health Organization (WHO) recognized four distinct entities of anaplastic large cell lymphoma (ALCL) including ALK-negative primary cutaneous ALCL, breast implant-associated ALCL, systemic ALK-negative (ALK(–)) or ALK-positive (ALK(+)) ALCL. Systemic ALCL, first described in 1985 by H. Stein, is considered as a mature T-cell lymphoma with an activated phenotype as all neoplastic cells display a constant strong expression of the CD30 molecule, a member of the tumor necrosis factor (TNF) receptor superfamily, normally expressed in activated lymphocytes [30,31]. This peripheral T cell lymphoma accounts for 2–8% of non-Hodgkin lymphomas in adults and approximately 10–30% of lymphomas in children. Extranodal involvements, in skin, liver, and gastrointestinal localizations, are common. ALK(+) ALCL is considered as a peripheral T cell lymphoma due to the expression of mature and activated T cell markers as well as the largely peripheral presentation of the tumors. However, ALK (+) ALCL also shares characteristics with an immature T cell. This therefore suggests that the final form in which ALK (+) disease occurs may mask its natural history [32,33,34,35]. ALK(+) ALCL by definition overexpresses an ALK fusion gene, typically t(2;5)(q23;35) (80% of cases) which fuses the 3′-part of the *ALK* (Anaplastic Lymphoma Kinase) gene on chromosome 2p23 with the 5′-part of the nucleophosmin (*NPM1*) gene on chromosome 5q35, resulting in an NPM-ALK chimeric protein with constitutive tyrosine kinase activity [36].

Nucleophosmin (NPM) is a ribonucleoprotein, encoded by the *NPM1* gene, located on chromosome 5. NPM1 is a multifunctional protein which has the capacity to bind nucleic acids and acts as a chaperone in the transport of pre-ribosomal particles from the nucleus to the cytoplasm. Thus, NPM1 is a bidirectional nuclear shuttle; its structure contains a bipartite nuclear localization signal. It is involved in ribosome maturation and export and centrosome duplication [37]. In addition, NPM plays a role in DNA repair, transcription, and regulation of genomic stability [38]. Of note, the reciprocal fusion product (ALK-NPM) is not expressed in ALCL and thereby is not pathologically relevant [39].

**Figure 2:**
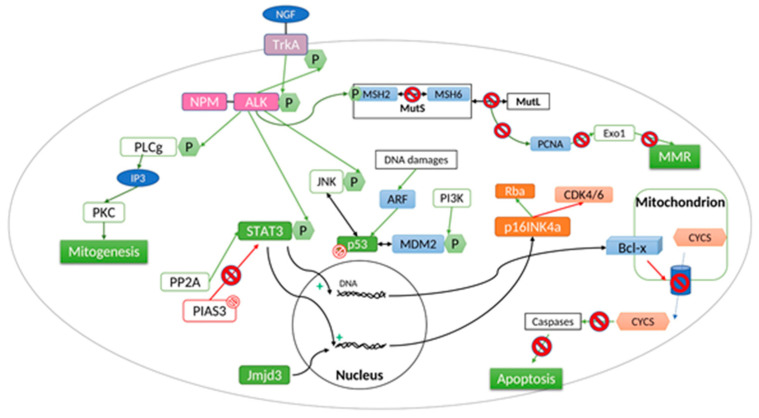
ALK-independent resistance mechanism.

NPM-ALK is the most common translocation in ALK(+) ALCL suggesting that these two genes are predisposed to translocation events in mature CD4(+) lymphocytes [33] and/or the NPM translocation partner provides a selective oncogenic advantage to incipient immortalized cells compared to other fusion proteins. The chimeric NPM-ALK protein is composed by the N-terminal portion of NPM (117 amino-acids) and the entire ALK cytoplasmic domain, its catalytic portion. NPM-ALK exists under two different forms, homodimer and heterodimer. Thanks to oligomerization domains, NPM-ALK dimerizes with wild type NPM. This form is located in the nucleus as wild type NPM brings the nucleus localization sequence to the protein. The homodimer form remains in the cytoplasm. Overexpression of NPM-ALK in the cytoplasm activates caspases (3 and 7) and leads to cell apoptosis. The N-terminal region of NPM contains the dimerization domain essential to autophophorylation of the NPM-ALK chimera protein as this domain mimes ligand-dependent aggregation and allows the transphosphorylation of NPM-ALK. Thus, the fusion protein is constitutively activated by autophosphorylation. This over-activation leads to absurd triggering of a variety of intracellular signaling cascades. The main outcomes are growth factor independent cellular proliferation, transforming potential, and apoptosis resistance (Figure 2).

## 3. Downstream Signaling Pathways Activated by NPM-ALK

NPM-ALK causes diverse pathogenic signaling anomalies that are closely interconnected or overlapping [40]. The oncogenic fusion tyrosine kinase, mainly promotes, 3 key signaling pathways: (1) Janus kinase 3 (JAK3)-STAT3 intracellular pathway; (2) phosphoinositide 3-kinase (PI3K)-Akt pathway; (3) RAS-extracellular signal regulated kinase (ERK) pathway to promote cell cycle progression, survival, and proliferation. Activation of the phospholipase C-γ also contributes to NPM-ALK-mediated transformation.

## 4. STAT3

Signal transducer and activator of transcription (STAT) proteins are latent transcription factors that reside in the cytoplasm and are activated by the receptor-associated Janus tyrosine family of kinases (JAKs) in response to cytokine signaling. STAT3 is activated by phosphorylation of tyrosine 705, by an intrinsic tyrosine kinase activity or a receptor associated Jak-Tyr kinase [41]. Jak3 is constitutively activated by NPM-ALK but this interaction is not strictly required to enhance cell transformation. Once phosphorylated, there is a dimerization of two STAT and a nuclear transfer. In the nucleus, it binds DNA to enhance the transcription of growth factor or cytokine response genes. Activated nuclear STAT3 has been implicated in maintaining cell survival by controlling the transcription of apoptosis-regulating proteins (e.g., Cyclin D1, Bcl-X, Bcl-XL, survivin, and c-Myc) thus promoting NPM-ALK(+) ALCL survival. When dysregulated, STAT3 becomes mitogenic and antiapoptotic. STAT3 regulated molecules are essential and required for the maintenance of the ALK-mediated neoplastic phenotype of ALCL cells. Indeed, through the upregulation of the *DNMT1* gene transcription, STAT3 is responsible for repressing the expression of T-cell genes that are commonly not expressed in NPM-ALK(+) such as CD3ɛ, ZAP-70, LAT, and SLP-76, and also of microRNA responsible for the chemotherapy and target drug resistances [42,43,44]. Thus, in NPM-ALK(+) ALCL, the presence of this transcription factor is a negative prognostic factor.

Activated STAT3 confers a T-regulatory phenotype to NPM-ALK(+) lymphoma cells and potentially contributes to their ability to evade the host’s immune response. Indeed, the fusion protein enhances the expression of FoxP3, and the expression and secretion of IL10 and TGFβ. These three proteins are involved in immune regulation and immune suppression. Furthermore, NPM-ALK(+) lymphoma cells highly express the immunosuppressive cell-surface receptor PD-L1 also called CD274/B7-H1 which is physiologically expressed by immune cells and binds PD1, present on the surface of CD4+ and CD8+ lymphocytes. This interaction between these two molecules enhances the induction of peripheral T-cell tolerance to self-antigens during the immune response. This limits the immune response against microorganisms, avoids an over-reaction of the immune system and the appearance of autoimmune diseases. In oncogenic situations, it leads to immune evasion. In NPM-ALK(+) cells, PDL1 gene transcription is highly enhanced by STAT3 binding its promoter. This change of phenotype, mediated by the chimeric protein, via STAT3 transcription activation, allows the NPM-ALK positive tumor to escape the immune system [45] but is not associated with patient outcome [46].

In conclusion, the activation of STAT3 has been strongly implicated in the pathogenesis of NPM-ALK(+) lymphoma. Thus, the inhibition of STAT3 in NPM-ALK(+) cell lines, either through the overexpression of a dominant negative STAT3 construct or decreasing STAT3 expression using antisense oligonucleotides, resulted in decreased proliferation and the induction of apoptosis [41].

## 5. PI3K

Phosphoinositide 3-kinase (PI3K) is an enzyme that transforms phosphoinositides into phosphoinositidylinositol 3 phosphate (IP3). IP3 recruits AKT, a serine/threonine-specific protein kinase, via PDK1 (phosphoinositide-dependent kinase-1). AKT then activates mTOR, a serine/threonine kinase involved in cell proliferation, cell growth and angiogenesis. The NPM-ALK protein triggers this signaling pathway by interacting with the SH2/SH3 domains of the regulatory subunit (p85) of PI3K that activates the catalytic subunit (p110). Signaling through the PI3K pathway promotes cell survival and proliferation in NPM-ALK+ ALCL. Treatment of NPM-ALK(+) cells with PI3K inhibitors induced apoptosis and reduced proliferation [47].

## 6. Ras-Extracellular Signal-Regulated Kinase (ERK) Pathway

Ras proteins play a role in controlling the activity of several signaling pathways which regulate normal cell proliferation. Activation of the ERK1/2 complexes results in the potentiation of a number of other substrates which operate in multiple cell functions including proliferation, survival, migration, cell division and differentiation. The NPM-ALK fusion protein activates ERK through the activity of the mitogen-activated protein kinases (MAPK). In the context of NPM-ALK(+) ALCL, ERK1/2 are known to drive proliferation by promoting cyclin-dependent kinase 4 (CDK) activity and phosphorylation of the retinoblastoma protein, and maintaining viability by positively regulating the expression of anti-apoptotic factors (e.g., Bcl-xL). Thus, the ERK pathway is activated in NPM-ALK(+) ALCL cells and plays a central role in promoting cell proliferation and suppressing apoptosis in this cancer [48]. Treatment with the MEK1/2 inhibitor, U0126, was found to reduce proliferation and enhance apoptosis in ALK+ ALCL cell lines [49]. Two important downstream mediators of MEK/ERK signaling in NPM-ALK(+) ALCL are the serine/threonine kinase, mammalian target of rapamycin (mTOR), and the JunB transcription factor. Inhibition of mTOR expression or function was associated with down-regulation of anti-apoptotic proteins, including c-FLIP, MCL-1, and BCL-2 and induction of apoptosis suggesting that inhibition of mTOR represents a potential therapeutic strategy in NPM-ALK+ ALCL [50].

## 7. PLC-Gamma

NPM-ALK activates phospholipase C-gamma (PLC-γ) by tyrosine phosphorylation [51,52]. This phospholipase belongs to the SH2-containing signaling molecule group. In fact, it has been proved that PLC-γ binds the fusion protein on Tyr664, located in the C-terminal domain of NPM-ALK. In NPM-ALK(+) cells, complex formation of PLC-γ with the constitutively activated NPM-ALK leads to tyrosine phosphorylation of PLC-γ and, presumably, to activation of the protein’s catalytic domain. Moreover, NPM-ALK phosphorylates PLC-γ on Tyr783 and Tyr1254 leading to the production of inositol triphosphate (IP3). IP3 activates protein kinase C (PKC). Knowing that PKC plays an essential role in mitogenesis [53] and that Tyr 664 is essential for mitogenic signaling in NPM-ALK(+) cells, the PLC-γ pathway is an important downstream target of NPM-ALK that contributes to its mitogenic activity and is likely to be important in the molecular pathogenesis of ALCL. The fact that the single phosphotyrosine residue 664 is required to mediate a mitogenic signal by NPM-ALK in lymphocytes opens the possibility of clinical therapeutic interventions at the molecular level. Indeed, it has been previously shown that the activation of PLC-γ can be completely blocked by cell-permeative peptides representing the PLC-γ-SH2 binding site [51].

## 8. HSP90

The activity of the NPM-ALK oncoprotein is also dependent on the molecular chaperone, heat shock protein-90 (HSP90) [54]. This kind of protein is involved in maturation, folding and stability of numerous proteins, including ALK. This is also the case for ALK-fusion proteins as HSP90 interacts with the ALK catalytic domain. HSP90 promotes the stability of NPM-ALK. Treatment of NPM-ALK(+) ALCL cells with the HSP90 inhibitor, 17-Allylamino-Demethoxygeldanamycin (17-AAG), resulted in decreased NPM-ALK expression, most likely due to the targeting of this protein for proteasomal degradation, but also to cell cycle arrest and to the induction of apoptosis [55,56]. HSP90 has also been shown to be important for the activity of metalloproteinase 9 (MMP9) and invasion in NPM-ALK(+) cells [57]. HSP90 inhibition also decreased levels of the pro-survival serine/threonine kinase Akt, the cell cycle-associated proteins cyclin D1, cyclin-dependent kinase 4 (cdk4), and cdk6, as well as several other proteins in NPM-ALK+ ALCL. The treatment of NPM-ALK(+) ALCL cells with 17-AAG also resulted in decreased phosphorylation of the serine/threonine kinase Erk without affecting Erk levels [56].

## 9. MicroRNA

Micro-RNAs (miRNAs), a class of small noncoding RNA (18–22 nt), have emerged as molecules which regulate diverse cellular functions such as proliferation. They regulate the expression of their target proteins through binding at the 3′-UTR regions of mRNAs by translational inhibition usually resulting in translational repression or more rarely in mammalian cells in the degradation of the targeted mRNA. Deregulated miRNAs have been observed in many cancers including ALK(+) ALCL with tumor suppressor or oncogenic functions [43]. STAT3-mediated activation of several microRNA and both STAT3 and NPM-ALK contributed to microRNA epigenetic silencing in ALK(+) ALCL human cell lines and primary biopsies by up-regulating and recruiting DNA methyltransferase 1 (DNMT1) to miRNAs promoters [43,58]. NPM-ALK itself is targeted by miRNA-96 [59]. Thus, inhibition or ectopic expression of miRNAs might represent an alternative avenue to interfere with ALK signaling in ALCL. Indeed, in ALK(+) ALCL human cell lines, demethylating treatment, or ectopic expression of some microRNAs decreased protein target levels and sensitized the tumoral cells to chemotherapy (miR29a/Mcl1 [60]) potentiating the action of crizotinib (miR7-5p/Raf1 [61]) or disadvantaging crizotinib-resistant NPM-ALK(+) cell growth (miR150/MYB [44]). In addition, microRNAs inhibition represents an alternative avenue to interfere with ALK signaling in ALCL (miR-17~92 [58]). Interestingly, we have observed that downregulation of miRNA-125b or overexpression of protein targets of miR-497 could be used as a biomarker for early relapse in human ALK(+) ALCL primary biopsies [62,63]. MicroRNAs could be a therapeutic target for the development of future treatments for ALK(+) ALCL.

## 10. Targeting Oncogenic ALK in Anaplastic Large Cell Lymphomas

The first common line of treatment in ALCL is a polychemotherapy, an anthracycline-containing regimen, usually CHOP which can be associated with radiotherapy in some cases. If patients are resistant or bad responders to chemotherapy, oncogenic ALK action can also be suppressed by small molecule inhibitors of ALK tyrosine kinase activity (TKI). Immunotherapy or molecules impairing NPM-ALK signaling pathways are being evaluated. Thus, the emergence of drug resistance led to the development of the first-, second-, third-, and fourth-generation ALK TKI. All are well studied and used in the treatment of EML4-ALK(+) lung cancer. However, the only one currently used in ALCL is Crizotinib, belonging to the first generation.

## 11. Chemotherapy

Since its discovery in 1974 [64], CHOP, composed of clophosphamide (Endoxan™), an alkylating agent; Hydroxyadriamycine (Adriblastine™), an intercalating agent; Oncovin™ (Vincristine), a vinca alkaloid, i.e., an agent which depolymerizes microtubules and which also interferes with amino acids, and Prednisone (Cortancyl™), a corticosteroid, is used in the treatment of lymphomas, including ALCL. Thus, CHOP or a CHOP-like regimen remains the standard treatment for ALK(+) ALCL, with a 5-year overall survival rate of 70–90% possibly due to the young age of the patients (median age 35 years) [65]. Indeed, the integration of etoposide, a DNA topoisomerase inhibitor II, in the primary treatment may be associated with significant improvements [66]. Moreover, vinblastine, which inhibits microtubule formation, enhances cell cycle arrest and then apoptosis, lags the occurrence of ALCL relapses when it is taken during polychemotherapy and during one year after. However, it does not decrease the risk of failure. For low-risk patients, the outcome is similar to polychemotherapy. As it is less toxic than CHOP, it could be used for this type of patient [66,67]. Recently, a GDPT regimen, i.e., GDP added to thalidomide, has shown to be a new promising approach to treat patients with relapse and refractory PTCL including ALK(+)ALCL [68]. Indeed, GDP, composed of Gemcitabine (an antimetabolite), Cisplatin (an alkylating agent) and Prednisone, was previously used to treat relapsed or refractory patients affected by Peripheral T-cell lymphoma (PTCL), including ALCL. IGDPT displays better outcomes than CHOP in PTCL [69].

## 12. Immunotherapy

cAC10, also called SGN30, is an antagonist of the CD30 antigen (Lymphoid activation antigen CD30, Ki-1 antigen). cAC10 is a chimeric anti-CD30 monoclonal antibody that is derived from the fusion of the variable heavy and light region of the murine anti-CD30 antibody AC10, with the constant γ-heavy and κ-light region of the human immunoglobulin. Administration of SGN-30 is safe, with modest clinical activity in patients with Hodgkin’s lymphoma (HL) or ALCL [70,71]. Monomethyl auristatin E (MMAE) is a synthetic derivative of dolastatin 10, a cytostatic pseudopeptide isolated from the marine shell-less mollusk *Dorabella auricularia*. MMAE induces cytostasis, tubulin-dependent GTP hydrolysis, and polymerization. Brentuximab vedotin is an antibody-drug conjugate consisting of the cAC10 monoclonal antibody and the MMAE cytotoxic agent. Brentuximab binds CD30, then the whole complex is internalized and trafficked to lysosomes. MMAE is released and disrupts the microtubule network which leads to cell cycle arrest in the G2/M phase and then to apoptosis [72]. This leads to tumor regression and durable objective responses associated with moderate adverse effects. Brentuximab is used to treat relapsed or refractory Hodgkin’s lymphomas and ALCL [73].

NPM-ALK promotes immune evasion by inducing the expression of the immunosuppressive cell surface protein, programmed death ligand 1 (PD-L1), through the activation of the STAT3 pathway [74] as well as a signalosome containing GRB2/SOS1, which activates the MEK-ERK and PI3K-AKT signaling pathways. Both STAT3 and GRB2/SOS1 signaling ultimately induce PD-L1 expression through the action of transcription factors IRF4 and BATF3 which directly regulate PD-L1 transcription [75]. Furthermore, all ALK+ ALCL biopsied tissues exhibited a strong STAT3 and IRF4 immunostaining in correlation with PD-L1 expression levels [75,76]. Interestingly, deleted PD-L1 expression in the tumor cells, or treatment of the culture with nivolumab, an anti-PD1 antibody used in clinical practice, restored T-cell activation [75,76]. Thus it was clearly demonstrate that a prolonged response can be achieved with anti-PD1 therapy in patients with NPM-ALK+ ALCL refractory to chemotherapy and ALK inhibitors [76] or after allogeneic hematopoietic stem cell transplant [77] and in patients with relapses [78]. All these reports provide a solid rationale for targeting programmed death 1 (PD-1/PD-L1) in a subset of relapsed/refractory NPM-ALK(+) ALCL.

## 13. Vaccination

The immune system helps to both maintain cancer and kill tumor cells. A specific anti-tumor vaccination could increase the quality of this immune response against malignant cells. With this objective in mind, anti-tumor vaccination strategies are based on the identification of one or more antigens which can be used to stimulate the immune system.

Studies on the immunogenicity of ALK in humans have shown that pediatric patients with ALK(+) ALCL developed a humoral immune response against ALK [79]. The titer of these autoantibodies titer is inversely correlated with the risk of relapse of ALK(+) ALCL [80]. Moreover, persistent anti-ALK antibody titers at the end of chemotherapy indicated protection against relapse ALK(+) pediatric and adolescent ALCL patients [81]. Spontaneous CD8(+) T-cell responses, against NPM-ALK can also be detected in a high proportion of patients. The antibodies produced by ALK(+) ALCL patients were mostly directed against the C-terminus of ALK protein. Furthermore, the ALK-kinase region and the C-terminus of the ALK protein contains immunodominant peptides inducing a CD8(+) T-cell response in humans [82] and were detected by antibodies of ALK-positive ALCL patients [83]. Moreover, using autologous dendritic cells expressing NPM-ALK oncogene as antigen-presenting cells for T cell stimulation NPM-ALK-specific CD8^+^ T cell responses were also detected in the sera of five ALK(+) ALCL patients in association with an anti-ALK antibody response [84]. The epitopes identified should now be further evaluated as possible targets for novel therapeutic strategies such as multi-epitope vaccination or direct targeting by engineered intracellular antibodies. Thus, ALK appears as an ideal oncoantigen for potential tumor vaccination and the monitoring of NPM-ALK-specific immune responses during therapy will provide new insights into the interplay between ALCL and the immune system.

## 14. Anti-ALK

The pharmaceutical industry took a long time to consider the idea of targeting ALK in ALCL as ALCL is an orphan disease. The discovery of EML4-ALK in non-small-cell lung cancer (NSCLC) opened the research domain of “anti-ALK” drugs. Nowadays, different classes of ALK inhibitors are available and used exclusively for NSCLC. Indeed, significant toxicities of the newest classes explain the lag of their use on ALK(+) ALCL patients, which are mostly children.

## 15. Crizotinib, The First Generation Drug

The presence of ALK fusion proteins and the constitutive ALK tyrosine kinase activity represents a therapeutic target in all malignancies with *ALK* rearrangements including ALCL. Furthermore, considering that ALK is not widely expressed in adult tissue, few toxic effects might be expected from treatments blocking ALK function. The first ALK inhibitor introduced in the treatment of ALK-dependent tumors, more specifically in NSCLC was the Pfizer compound PF-02341066 (Crizotinib, Xalkori). This ALK tyrosine kinase inhibitor is a small-potent oral molecule, which also inhibits mesenchymal–epithelial transition factor (c-MET) and c-ROS kinases [85,86]. Crizotinib potentially stops the growth of the tumor by blocking the tyrosine kinase activity of ALK [85].

Crizotinib, initially designed as a MET inhibitor in 2007, was first approved in 2011 by the US Food and Drug Administration (FDA) for the treatment of locally advanced or metastatic ALK-rearrangement NSCLC. Metastatic NSCLC treated with crizotinib showed an objective response rate close to 70% and tumor stabilisation or shrinkage in 90% of patients [87]. Similarly, crizotinib also showed therapeutic responses in ALK-fusion-positive inflammatory myofibroblastic tumor (IMT) patients [88] and paediatric patients with ALCL and IMT [89]. Nowadays, several trials on ALK(+) ALCL patients are in progress around the world. A phase 1 trial evaluating the combination of Crizotinib and chemotherapy on children affected by relapsed or refractory ALCL (and other ALK related tumors) is ongoing since 2012 (NCT01606878). Another trial (NCT01979536–phase 2) is comparing the outcomes of Crizotinib and Brentuximab vendotin treatment in combination with multi-agent chemotherapy in patients with newly diagnosed stage II-IV ALCL. NCT02419287 studies the effects of Crizotinib alone on relapsed ALK(+) lymphomas in an adult population. However, as for ALK(+) lung cancer, some ALK(+) ALCL patients do not respond to crizotinib or an acquired resistance occurs several months following an initial response. This is not surprising, as it has already been observed clinically with other tyrosine kinase inhibitors, such as imatinib in chronic myeloid leukaemia [90].

Resistance to crizotinib was originally reported in NSCLC [91,92] and inflammatory myofibroblastic tumor [93] followed by neuroblastoma [89] and ALCL [94]. Based on the knowledge stemming from NSCLC, several mechanisms of resistance to crizotinib have been described defined as ALK-dependent or non ALK-dependent. Emergence upon crizotinib treatment of mutations within the ALK kinase domain include F1174L, F1174C, C1156Y, L1196M, I1171T, G1202R, G1269A, S1206Y, and the gatekeeper mutation L1196M [95]. These resistance mutations can be divided into two main categories. The first one includes mutations of the residues that enable direct contact with the inhibitor, thus impairing its binding due to steric hindrance. The second class includes mutations of residues at distance from the inhibitor-binding site that promote conformational changes increasing ALK kinase activity [93,96]. In EML4-ALK(+) NSCLC, gain in ALK copy number, loss of ALK gene rearrangement and engagement of other cell signaling pathways mediated by increased phosphorylation of EGFR, amplification of KIT or KRAS mutations have also been implicated in the development of acquired resistance to crizotinib. In this scenario, malignant cells use alternative ALK independent oncogenic mechanisms [97]. In NSCLC, the most common site of relapse after crizotinib treatment is the central nervous system (CNS) as the consequence of P-glycoprotein-mediated efflux which is responsible for the poor accumulation of the drug in the CNS [98]. By contrast NPM-ALK+ ALCL that became non-responsive to crizotinib revealed only ALK dependent mechanisms through mutations in the ALK kinase domain. Thus, in ALK+ ALCL disease progression was primarily caused by resistance to the inhibitor, rather than gain ALK independence [94].

## 16. Second- and Third-Generation of ALK Inhibitors

Despite the success of crizotinib’s therapeutic treatment, there was a need to conceive new drugs with better brain penetrance, higher specificity and targeting a broader set of resistance mutations. Acquired crizotinib resistance mediated by mutations in the ALK kinase domain can now be overcome by second-generation ALK inhibitors and ALK gene mutations may impact clinical decisions. Indeed, some mutations, such as the kinase pocket gatekeeper mutation at L1196, may be overcome by most of the second- and third-generation drugs, but other mutations are particularly cross-resistant and remain sensitive to only a subset of the available inhibitors. Indeed, clinical studies have shown that most but not all patients with ALK+ NSCLC with resistance to crizotinib respond to two drugs, ceritinib (Novartis, LDK 378, Zykadia, approved by the FDA in 2014) and Roche’s alectinib (CH5424802, AF802) which was FDA approved in 2015 for therapy in crizotinib-resistant patients [99,100] They provided good brain penetrance and a significant progression-free survival benefit against chemotherapy and/or crizotinib in the first line of treatment as demonstrated in the ASCEND-4 and ALEX trials [101]. Brigatinib from Ariad and Takeda, also an inhibitor of mutated EGFR, was the latest second-generation ALK inhibitor approved in 2017 by the US FDA for ALK(+) NSCLC. This drug is very similar to alectinib in efficacy, while being active against some resistant mutations such as the common G1202R mutation that provides resistance to alectinib. Other second generation ALK inhibitors are currently under clinical investigation: AP26113 developed by Ariad, a dual ALK and EGFR inhibitor, currently in phase I/II clinical trial (NCT01449461) and ASP3026 by Astelle Pharma, structurally related to NVP-TAE684, which is also undergoing phase I trials (NCT01284192 and NCT01401504) [102]. As expected, interest in new drugs that may be able to overcome crizotinib resistance is growing fast. In ALK+ ALCL cell lines, resistance to both first-generation crizotinib and second-generation alectinib was a result of the emergence of additional mutations beyond the gatekeeper mutation [103] and some mutations, such as I1171, are particularly cross-resistant [94,104].

To optimize the therapy of patients who have developed resistance to crizotinib, DNA sequence analysis of the ALK gene in individual patients to identify mutations to the specific ALK inhibitors could be useful. The same analysis should be applied to tumors that fail to respond to the second-or third-generation ALK inhibitors. A third-generation inhibitor, lorlatinib, remains active against a broad range of known ALK mutations in preclinical models of EML4-ALK+ NSCLC and neuroblastoma [104].

## 17. Investigational Associations with ALK-Inhibitors in Anaplastic Large-Cell Lymphoma

To act on ALK-inhibitor resistance the new strategy involves a combinational therapy using inhibition of ALK associated with another drug. This strategy is still under preclinical/clinical investigation but could overcome ALK inhibitor resistance. Accordingly, a phase 1 clinical trial to test crizotinib plus chemotherapy in children has been performed (NCT01606878). Moreover, promising results have been obtained with the HSP90 inhibitor, such as NVP-AUY922/Luminespib (Novartis) which is currently tested in EML4-ALK(+) NSCLC patients as a single agent or in combination with the ALK inhibitor LDK378 (NCT01124864, NTC01752400, NTC01772797). NVP-AUY922 could also potentially be applied for the management of ALK(+) ALCL in naïve patients and in patients who have relapsed through acquisition of resistance. Indeed, NVP-AUY922 induced growth inhibition in the parental but also in the resistant ALK(+) ALCL cell line KARPAS-299 [103]. Thus, a phase two trial (NCT02572453) including Onalespib/AT-13387 (Astex Therapeutics), a HSP90 inhibitor, is ongoing in patients with relapses/refractory ALK(+) ALCL. Other combinations may also contain inhibitors of key cell signaling pathways downstream of NPM-ALK and involved in ALK oncogenicity [105]. STAT3 is another attractive candidate, as it is a key NPM-ALK–activated gene transcriptional modulator [106]. However, currently available STAT3 inhibitors have serious limitations because they are poor tissue-penetrating oligonucleotides or small molecules with a fairly low specificity [107]. Given the role of DNMTs in inhibiting tumor suppressors in ALK+ ALCL such as miRNA, they are also potential therapeutic co-targets with ALK in crizotinib resistant ALK(+) ALCL cells as previously shown [42,44]. In EML-ALK(+) NSCLC, while targeted therapies provide limited duration of responses characterized by the development of resistance, PD-1 inhibitors achieve more modest but durable responses [108]. Therefore, combining crizotinib and a PD-1 inhibitor (such as nivolumab, Bristol Myers Squibb) may improve the long-term outcome in ALK- translocation positive NSCLC. Protein degradation by proteolysis-targeting chimera (PROTAC) is a bifunctional-hybrid molecule that includes PROTACs a ligand targeting a protein of interest, a ligand targeting an E3 ligase and a connecting linker. The aim is inhibiting the target to induce its proteasomal degradation by ubiquitin-proteasome system (UPS) which is the primary intracellular mechanism for destruction of damaged proteins [109]. Since 2018, a PROTAC is being tested on ALK fusion proteins. Pomalidomide/Imnovid^®^ (a derivative of Thalidomide, Celgene), which acts as an anti-angiogenic and immunomodulatory agent, is fused to an ALK inhibitor, Ceritinib in combinaison with an ALK PROTACs (degraders). The latter can inhibit phosphorylation of ALK and STAT3 in the NPM-ALK(+) ALCL cell line, SU-DHL1 [110].

## 18. Conclusions

Each ALK inhibitor exhibits its molecular response and monitoring for emerging resistance mutations is crucial for an effective treatment outcome. As for patients with EML4-ALK(+) NSCLC, with crizotinib resistant mutations, NPM-ALK(+) ALCL patients could benefit in the years to come from second- and third-generation ALK inhibitors alone or in combination with inhibitors of key downstream cell signaling pathways or key regulators of ALK tyrosine kinase activity. Immunotherapy strategies are under investigation in NPM-ALK(+) ALCL chemotherapy-resistant cases. However, chemotherapy remains a viable option in ALCL patients with ALK translocations as it may be more effective than other non-chemotherapy compounds.

## Figures and Tables

**Table 1:** ALK rearrangements in human malignancies.

Cancer Type	ALK Fusion Partner
Anaplastic Large Cell Lymphomas	NPM1 (5q35.1)
	TPM3 (1q21.3)
	ATIC (2q35)
	TFG (3q12.2)
	TRAF1 (9q33.2)
	CLTC (17q23.1)
	RNF213 (17q25.3)
	TPM4 (19p13.1)
	MYH9 (22q12.3)
	MSN (Xq12)
	Additional rare rearrangements
Non-Small Cell Lung Cancer	EML4 (2p21)
	TPR (1q31.1)
	CRIM1 (2p22.2)
	STRN (2p22.1)
	TFG (3q12.2)
	HIP1 (7q11.23)
	PTPN3 (9q31)
	KIF5B (10p11.22)
	KLC1 (14q32.3)
	CLTC (17q23.1)
Inflammatory Myofibroblastic Tumor	TPM3 (1q21.3)
	RANBP2 (2q13)
	ATIC (2q35)
	SEC31A (4q21.22)
	CARS (11p15.4)
	PPFIBP1 (12p11)
	CLTC (17q23.1)
	TPM4 (19p13.1)
Diffuse Large B Cell Lymphomas	RANBP2 (2q13)
	EML4 (2p21)
	SEC31A (4q21.22)
	SQSTM1 (5q35)
	NPM1 (5q35.1)
Renal Cell Carcinoma	VCL (10q22.2)
	TPM3 (1q21.2)
	EML4 (2p21)
	STRN (2p22.2)
Colorectal Cancer	EML4 (2p21)
	WDCP (2p23.3)
Breast cancer	EML4 (2p21)
Esophageal cancer	TPM4 (19p13.1)
Ovarian cancer	FN1 (2q35)
Renal Medullary Carcinoma	VCL (10q22.2)
